# Outbreak of H7N8 Low Pathogenic Avian Influenza in Commercial Turkeys with Spontaneous Mutation to Highly Pathogenic Avian Influenza

**DOI:** 10.1128/genomeA.00457-16

**Published:** 2016-06-16

**Authors:** Mary Lea Killian, Mia Kim-Torchetti, Nichole Hines, Sam Yingst, Thomas DeLiberto, Dong-Hun Lee

**Affiliations:** a National Veterinary Services Laboratories, Veterinary Services, U.S. Department of Agriculture (USDA), Ames, Iowa, USA; b Animal Disease Diagnostic Laboratory, Purdue University, West Lafayette, Indiana, USA; c USDA Wildlife Services National Wildlife Research Center, Fort Collins, Colorado, USA; d USDA Agriculture Research Service, Southeast Poultry Research Laboratory, Athens, Georgia, USA

## Abstract

Highly pathogenic avian influenza (HPAI) subtype H7N8 was detected in commercial turkeys in January 2016. Control zone surveillance discovered a progenitor low pathogenic avian influenza (LPAI) virus in surrounding turkey flocks. Data analysis supports a single LPAI virus introduction followed by spontaneous mutation to HPAI on a single premises.

## GENOME ANNOUNCEMENT

In January 2016, the Indiana State Board of Animal Health (BOAH) veterinarians were notified of increased mortality at a commercial turkey operation (1). Decreased water consumption and recumbent turkeys with pulmonary congestion and edema were reported in one of six barns. Three days following the onset of clinical signs, mortality increased from 100 to 800 birds within 24 h. Birds in the five other barns on the index premises showed no clinical signs. Turkeys from the affected barn tested positive for H7 by PCR, and partial sequencing confirmed H7N8 highly pathogenic avian influenza (HPAI). Additional samples collected within a 10 km zone surrounding the index farm identified eight additional H7-positive premises, none with clinical signs, and partial sequencing confirmed H7N8 low pathogenic avian influenza (LPAI).

This is the first detection of an H7N8 HPAI virus in any species. The virus is not related to the H5 HPAI viruses that caused outbreaks in the United States from 2014 to 2015. Based on full genome sequences representing seven different premises, the index H7N8 HPAI virus and subsequent H7N8 LPAI viruses are of North American wild bird lineage and are highly similar to each other across all eight genes (excluding the multi-basic amino acid insertion at the cleavage site responsible for the mutation to HPAI). The H7N8 HPAI virus contained a 9-nucleotide insertion at the hemagglutinin gene cleavage site translating to basic amino acids (PKKRKTR*G).

The H7N8 virus is also highly similar across six of eight genes (except nucleoprotein and matrix) to a recent H7N8 LPAI virus isolated from a hunter-harvested male lesser scaup (after-hatch year) in Kentucky during November 2015 as part of the national wild bird surveillance effort. This indicates that reassortment likely occurred prior to virus introduction in turkeys in Indiana. However, mutation to HPAI likely occurred during replication of the virus in poultry.

In general, influenza A viruses circulate in wild waterfowl, and the H5 and H7 subtypes have the potential for mutation from LPAI to HPAI which requires the acquisition of a multibasic cleavage site (http://www.oie.int/fileadmin/Home/eng/Health_standards/tahm/2.03.04_AI.pdf). Mechanisms for acquiring multibasic amino acids include (a) insertion of basic amino acids from codons duplicated at the cleavage site during transcription (2, 3), (b) accumulation of basic amino acids by gradual mutation resulting in amino acid substitutions, and (c) nonhomologous recombination resulting in the insertion of a foreign nucleotide sequence adjacent to the hemagglutinin (HA) gene cleavage site (4).

Additional genetic analysis of eight H7N8 viruses for which whole-genome sequences were obtained suggests a single introduction event was followed by lateral/secondary spread and provides potential insights into virus evolution and the epidemiology of this outbreak. Further studies are required to determine the mode of acquisition of the multibasic amino acid insertion at the HA gene cleavage site for this virus.

### Nucleotide sequence accession numbers.

The genome sequence of HPAI A/turkey/Indiana/16-001403-1/2016(H7N8), LPAI A/turkey/Indiana/16-001573-2/2016(H7N8), and LPAI A/turkey/Indiana/16-001574-7/2016(H7N8) are deposited in GenBank under accession numbers KU558903 to KU558910, KU585905 to KU585912, and KU585913 to KU585920, respectively.
